# Iodination of terminal alkynes using KI/CuSO_4_ – A facile method with potential for radio-iodination

**DOI:** 10.1016/j.tetlet.2019.02.041

**Published:** 2019-03-28

**Authors:** Trevor Ferris, Laurence Carroll, Ronnie C. Mease, Alan C. Spivey, Eric O. Aboagye

**Affiliations:** a Comprehensive Cancer Imaging Centre, Department of Surgery & Cancer, Hammersmith Campus, Imperial College, London W12 0NN, UK; b Russell H. Morgan Department of Radiology and Radiological Sciences, Johns Hopkins Medical Institutions, Baltimore, MD 21231, USA; c Department of Chemistry, Molecular Sciences Research Hub, 80 Wood Lane, White City Campus, Imperial College, London W12 0BZ, UK

## Abstract

Herein, we report an efficient new method for the iodination of terminal alkynes using stoichiometric KI and CuSO_4_ in a mix of acetonitrile and acetate buffer that holds promise for further development into a method for radio-iodination.

## Highlights

• Facile copper-catalyzed terminal iodination of alkynes, demonstrated on multiple examples.
• Demonstrated on a complex peptide, in high yield.
• Applicable to radioiodination work, with iodine-125 used.

## Graphical abstract

Herein, we report an efficient new method for iodination of terminal alkynes using stoichiometric KI and CuSO_4_ in acetate buffer that holds promise for further development into a method for radio-iodination.

**Figure:**
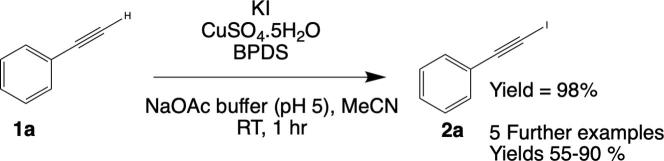

## Introduction

**Fig. 1:**
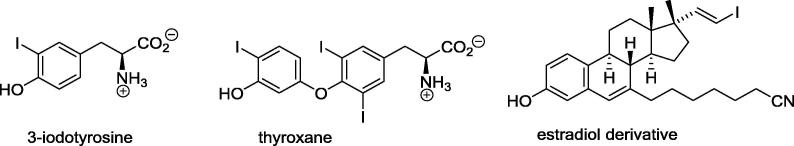
Clinically used iodo-tracer probes.

Radio-iodinated probes are useful in the context of biological research, diagnostic imaging and radiotherapy using ^125^I, ^123/4^I and ^131^I, respectively [1]. As alkyl iodides are relatively labile under physiological conditions, aryl and occasionally alkenyl iodides are often preferred for such purposes (Fig. 1) [1], [2].

The synthesis of these radio-labelled molecules is usually achieved by electrophilic iodo-dehydrogenation (*i.e.* C_sp2_-H → C_sp2_-I) or iodo-demetallation (*i.e.* C_sp2_-M → C_sp2_-I) reactions of appropriate aromatic/alkenyl precursors using carrier-added molecular iodine or iodide salts in the presence of an oxidant. The former reactions are generally performed on electron rich aryl precursors and are prone to poor regio- and chemoselectivity [3], whereas the latter reactions require the preparation of the appropriate aryl metal precursors (*e.g*. Ar-SnR_3_) which can be time consuming and involve toxic reagents (*e.g*. Cl-SnR_3_). Moreover, although this type of approach has been used extensively for the radioiodination of tyrosine containing peptides and proteins [4], the resulting probes often display poor metabolic stability [1]. The metabolic degradation of iodine-containing probes compromises their effectiveness and leads to an accumulation of iodine in the thyroid and stomach [5]. The activity/selectivity profile of these iodinated derivatives towards their *in vivo* targets also often differs significantly relative to the non-iodinated parent peptides and proteins [6], likely due to the steric and electronic perturbation that the iodine atom has on adjacent functionality e.g. the phenol moiety in the case of tyrosine based probes [7].

In view of this situation, we became interested in developing the iodination of terminal alkynes as an alternative approach to prepare radio-iodinated probes. In particular, we envisioned that this reaction could be highly chemoselective even in the presence of electron rich aromatic and heteroaromatic rings as well as electron rich alkenes. Additionally, such a protocol would introduce the iodine atom at a position unlikely to strongly sterically or electronically perturb adjacent functionality. It was anticipated that the iodoalkenyl products might also display improved metabolic stability relative to aryl and alkenyl analogues due to the greater strength of C_sp_-I bonds relative to C_sp2_-I bonds.

An ideal radiolabelling protocol would proceed rapidly under mild aqueous conditions (^123^I has t_1/2_ = 13 h), would avoid the use of carrier added molecular iodine to form an electrophilic iodine species due to its volatility/toxicity [8] and would be amenable to small scale preparations on an automated platform. Although the literature contains a number of protocols for the iododehydrogenation of terminal alkynes, none of them appeared well aligned to this aspiration [9], [10], [11], [12], [13].

## Results and discussion

Cognisant of the method of Tsai and co-workers [10]. in which iodination of terminal alkynes is achieved in aqueous solution using molecular iodine (2 equiv.), triethylamine (3 equiv.), tetrabutylammonium bromide (TBAB, 1 equiv.) and copper(I) iodide (1–2 mol%), we set out to explore the ability of combinations of various iodine/iodide sources and copper salts to effect the iodination of ethynylbenzene **1a** as a test substrate. In all experiments we used sodium acetate buffer solution at pH 5 as the medium with bathophenanthrolinedisulfonic acid (BPDS, 1 equiv.) added to solubilise copper species in this solution (Table 1).

**Table 1:** Reaction of alkyne **1a** with various iodine and copper sources to give iodoalkyne **2a.**

**Figure:**
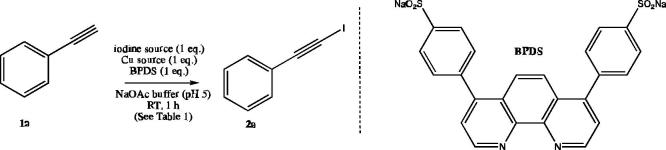

Entry	Source of Iodine	Source of Copper	Yield 2a
1	KI	CuSO_4_·5H_2_O	>99%
2	KI	CuSO_4_·5H_2_O (0.2 eq.)	45%
3	KI	Cu Wire	10%
4	I_2_	CuSO_4_·5H_2_O	25%
5	NIS	CuSO_4_·5H_2_O	0%

It pertained that a combination of potassium iodide (1 equiv.) and copper(II) sulfate (1 equiv.) was able to promote the reaction to give iodoalkyne **2a** in essentially quantitative yield within 1 h at room temperature (Entry 1). Reducing the amount of copper(II) sulfate to just 0.2 equiv. reduced the yield to 45% under otherwise identical conditions (Entry 2). Switching the copper source to copper wire reduced the yield further to 10% (Entry 3). Use of molecular iodine in place of potassium iodide gave just 25% yield (Entry 4) and use of *N*-iodosuccinimide in place of potassium iodide failed to effect any conversion to product **2a** (Entry 5). Although we have not performed any detailed mechanistic studies, these findings suggest that the reaction likely involves oxidation of the potassium iodide by the copper(II) salt to give an electrophilic iodine species which effects iododehydrogenation of the acetylene, probably *via* a copper acetylide.

With efficient conditions established (*cf*. entry 1, Table 1), we then moved on to look at the scope of the method using a series of aromatic alkynes and a reaction duration of just 10 min (Table 2).

**Table 2:** Reaction of aryl alkynes **1a-f** to give iodoalkynes **2a-f**.

**Figure:**
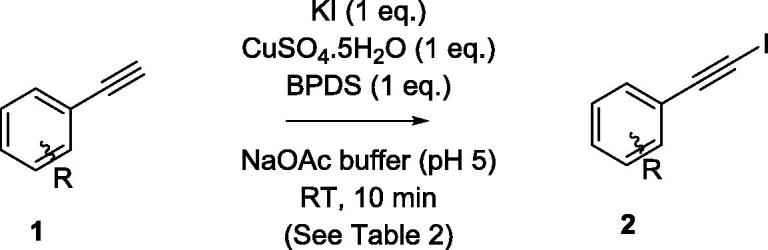

Entry	Alkyne	Iodoalkyne	Isolated Yield
1			98%
2			60%
3			82%
4			80%
5			55%
6			90%

Good to excellent yields were recorded in all examples (55–98%), with no dramatic variation according to the presence of electron-withdrawing or donating groups on the aryl ring (Entries 1–6).

**Graph 1:**
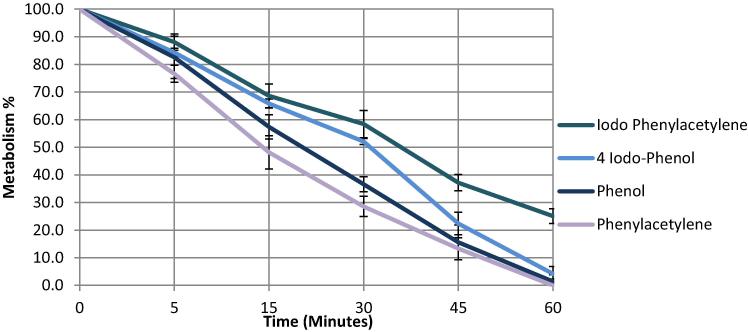
Summary of the metabolic profiles of each of the four test compounds used over 60 min.

Next, we performed an experiment to qualitatively compare the metabolic stability of 4-iodophenol *vs*. (iodoethynyl)benzene (**2a**). Phenol and ethynyl benzene (**1a**) were used as controls. Each compound was incubated with human liver microsomes according to the method of Jia and co-workers [14]. The concentration of each compound was followed by HPLC (see ESI for further details). Three repeats were conducted for each time point: 0 min, 5 min, 15 min, 30 min, 45 min and 60 min (Graph 1).

It is clear that over the period of 60 min, which corresponds to the duration of a routine human PET scan, the terminally iodinated alkyne substrate is significantly more stable than either the iodinated aromatic species (25% versus 5% remaining at 60 min) or the non-iodinated analogues: phenol and ethynylbenzene. Whilst it is not certain that the degradation/metabolised species generated are specifically related to the removal of the iodine atom from the parent compound, it is likely given the lack of other functionality in these simple test compounds. We tentatively conclude that iodoalkynes have promising metabolic stability and therefore radio-labelled congeners may hold promise for imaging applications.

**Scheme 1:**
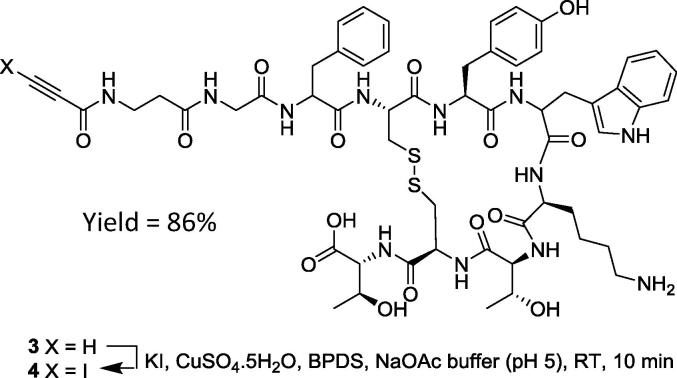
Synthesis of iodoethynyl βAG-TOCA derivative **4**.

Lastly, we looked to prepare an iodoalkyne derivative of the peptide βAG-TOCA using our new method. βAG-TOCA is an analogue of octreotate, a compound that has been shown to exhibit high agonist binding affinity to the somatostatin receptor SSTR-2 expressed in neuroendocrine tumours (NETs). Previous work undertaken by us [15], described a novel radiotracer, 18F-FET-βAG-TOCA, for imaging SSTR-2 expression by Positron Emission Tomography (PET). Radio-Iodination of βAG-TOCA with ^131^I could provide an alternative tracer for monitoring patients with gastroenteropancreatic neuroendocrine tumours. Thus, using the same terminal alkynyl precursor **3** used in the preparation of FET-βAG-TOCA (*via* a click reaction with fluoroethyl azide), iodination to give product **4** was achieved by subjection to our standard conditions (Scheme 1).

Following purification by HPLC, I-βAG-TOCA **4** was obtained pure (R_t_ 8:1 min), with the expected molecular ion by MALDI-TOF MS (*m*/*z* = 1354) and displaying a weak stretch in the FT-IR at 1901 cm^−1^ corresponding to the C-I bond stretch (see ESI for chromatogram and spectra).

**Scheme 2:**
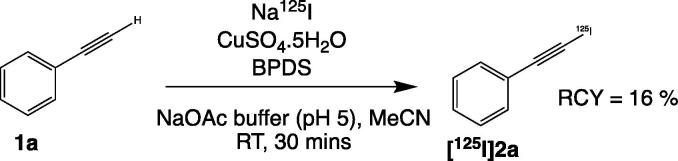
Radiosynthesis of **[^125^I]2a** from **1a.**

To confirm that this method of iodination would be applicable to radioiodination, we subsequently attempted to use the optimal conditions developed for non-radioactive iodine, with a form of radioactive iodine (Scheme 2). We chose ^125^I due to its long half-life for manipulation (t_1/2_ = 59.5 days) and because it is readily available in high specific activity. Reducing the amounts of the various reagents to be compatible with conditions regularly used for isotopic incorporation (see ESI for further details), and using Na^125^I (aqueous solution from Perkin-Elmer) of approximately 50 μCi per reaction, the reaction mixtures were left for 30 min at room temperature, followed by radio-HPLC and iTLC. The desired product peak was collected for accurate assessment of the radiochemical yield, which was found to be 16% of the radioiodinated-phenylacetylene derivative **[^125^I]2a**, as confirmed *via* both methods of analysis.

## Conclusion

We have described an operationally simple new method for the transformation of terminal alkynes into their iodinated congeners using stoichiometric KI and CuSO_4_ in the presence of BPDS in acetate buffer solution. The reaction durations are short (<10 min) and the yields generally high (55–98%, 7 examples). The reaction also proved successful for the introduction of ^125^I into a model substrate and for the preparation of an octapeptide: iodoethynyl βAG-TOCA **4**, suggesting the protocol displays high levels of chemoselectivity.

Given that preliminary experiments on (iodoethynyl)benzene (**2a**) indicate that this compound has superior stability towards degradation by human liver microsomes as compared to iodophenol, we believe that the method has potential *e.g*. for application in the preparation of radio-iodinated probes for imaging applications. Research towards this goal is ongoing in our laboratory.
