# Supplementary material for: Iodination of terminal alkynes using KI/CuSO4 – A facile method with potential for radio-iodination
Source: Tetrahedron Lett. 2019 Mar 28;60(13):936–9. doi: 10.1016/j.tetlet.2019.02.041 (PMC6562058; doi:10.1016/j.tetlet.2019.02.041)
Supplement: Supplementary data 1 [file mmc1.docx]

**Supplementary Information for:**

**Iodination of Terminal Alkynes Using NaI/CuSO_4_ – A Facile Method with Potential for Radio-iodination**

**Trevor Ferris,^a^ Laurence Carroll,^a,b^ Ronnie C Mease,^b^ Alan C Spivey^c^ and Eric O Aboagye*^a^**

*^a^Comprehensive Cancer Imaging Centre, Department of Surgery & Cancer, Hammersmith Campus, Imperial College, London, W12 0HS,UK.*

*^b^Russell H. Morgan Department of Radiology and Radiological Sciences, Johns Hopkins Medical Institutions, Baltimore, MD 21231, USA.*

*^c^Department of Chemistry, Molecular Sciences Research Hub, 80 Wood Lane, White City Campus, Imperial College, London W12 0BZ, UK*

*E-mail: eric.aboagye@imperial.ac.uk; Tel: +44 (0)20 8383 3759*

**Table of Contents**

General Procedures, Materials, and Instrumentation. S2 - S3

Synthesis Procedures and Characterisation. S4 - S5

NMR Spectra of Compounds. S7 – S12

Metabolic Experimental Procedure. S13

HPLC Procedure for Metabolism Studies. S13

Synthesis Procedure for Iodoethynyl βAG-TOCA. S14

HPLC Procedure for Iodoethynyl βAG-TOCA. S14

NMR & Mass Spectrometry Data for Iodoethynyl ΒAG-TOCA Derivative. S15

Radiolabelling Procedure for **2a** S16

References. S16

**General Procedures, Materials, and Instrumentation**

Reagents and solvents were purchased from Sigma-Aldrich Co. Ltd. (Gillingham, United Kingdom). Chemicals were not purified further than supplied.

All reactions were performed under mild conditions and normal atmospheric conditions.

Flash chromatography (FC) was always undertaken using silica gel (Sigma Aldrich 60Å F_254_ 230-400 mesh) petroleum ether and ethyl acetate mobile phase 95% to 5%. Thin layer chromatography was performed on Sigma Aldrich aluminium backed plated pre-coated with silica (0.2 mm, 60 F_254_) visualised with ultraviolet fluorescence λ = 254.

Semi-preparative HPLC was carried out using an Agilent 1100 series apparatus with Laura software (Ver 33.10.49). A Phenomenex Luna 5u C18(2), 100A,100 x 10 mm, 5-micron semi-preparative column with a flow rate of 3 mL/min was used for purification small molecules 1, 2, and 3. The following mobile phase system was used: solvent A, Water/Formic Acid (0.1%); solvent B Acetonitrile/Formic Acid (0.1%). 0-20 minutes 60% to 80% B, 20-25 minutes 80% to 60% B, 25-30 minutes 60% B. For compounds 4, 5 and 6, a Phenomenex Gemini 5u C18(2), 110A,100 x 10 mm, 5-micron semi-preparative column, Phenomenex SeurityGuard SemiPrep Cartridge Gemmini C18 x 10 x 10 mm, with a flow rate of 3 mL/min was used. The following mobile phase system was used: A 20% potassium phosphate buffer 20mM pH 7, B 80 % methanol, 30 minutes.

Nuclear magnetic resonance (NMR) spectroscopy was recorded at 400 MHz on a Bruker AV-400 instrument. Chemical shifts (δ_H_) are quoted in parts per million (ppm), referenced to the appropriate solvent peak. ^13^C NMR spectra were recorded on a 100 MHz on Bruker AV-400 instrument. Chemical shifts (δ_C_) are quoted in ppm. Acquisition software for the apparatus was ICON NMR 4.7.2 Build 30, TopSpin 3.2.

Fourier transform infrared (FTIR) spectroscopy was undertaken with a Perkin Elmer FTIR System Spectrum BX instrument and a Pike Miracle Anvil. Software set to 12 scans per sample between 400 and 4000 cm^-1^ wavenumbers.

For compounds 1-6, electron ionisation (EI) gas chromatography mass spectrometry (GC-MS) was undertaken with an Agilent Technologies 5975 inert XL Mass Selective Detector, 6890 N Network GC System with 7683B Injector. Phase: ZB-5MSplus, source temperature 230^o^C, run time 19.33 minute, 0-1 minute: 50^o^C, 1 min to 9.33 minutes ramp to 300^o^C and held. Software GCMS Mass Hunter: data analysis and acquisition.

For I-βAG-TOCA (ES-ToF) mass spectrometry was undertaken using an Acquity i-Class Waters LCT Premier mass spectrometer.

**Synthesis Procedures and Characterisation**

**General Procedure for the Synthesis of Iodinated Small Compounds**

# To acetonitrile (40 mL) was added the alkyne (2 mg). Bathophenanthrolinedisulfonic acid disodium salt hydrate (BPDS) (1 eq) was dissolved in purified water (100 µL) and added to the alkyne solution. The solution was vigorously stirred for 5 minutes. Finely ground potassium iodine (KI), (1eq) was added to the solution and vigorously mixed for a further 5 minutes. A solution of sodium acetate buffer (pH 5.0, 250 mM) was prepared. Copper (II) sulfate pentahydrate (1 eq, 5 µL) was mixed with the buffer and then added to the alkyne solution. Flash chromatography was then used to purify the compounds using a gradient mixture of petroleum ether and ethyl acetate. Solvents were then removed by rotary evaporator. Further purification was undertaken using reverse phase semi preparative HPLC as required. HPLC fractions were concentrated by rotary evaporator. Water and formic acid were removed by lyophilization, iodinated alkynes were then recovered.

Chemoselectivity was determined by NMR. Deprotonation was observed with the disappearance of a proton at 3.08 (s 1H) indicating binding of iodine to the alkyne. Furthermore, the peak at 10.05 (s, 1H) indicated that this proton has not been removed.

# (Iodoethynyl)benzene **2a** (Table 1, entry 1)^1^

^1^H NMR (400 MHz, DMSO-*d*_6_) δ 7.78 – 7.70 (m, 1H), 7.52 – 7.40 (m, 1H), 7.45 – 7.30 (m, 31), 7.23 – 7.13 (m, 1H).

^13^C NMR (101 MHz, DMSO-*d*_6_) δ 135.0, 130.0, 127.2, 126.9, 126.2, 120.2, 81.8, 78.8.

FTIR, 2100 w (C≡C stretch), 1599 s (C=C aromatic bend), 1537 m (C=C aromatic bend), 1486 m (C=C aromatic stretch), 754 s (C-H aromatic bend).

MS (EI^+^) (*m/z*): [M]^+^, 227.9, C_8_H_5_I requires [M]^+^, 227.9

1-(Iodoethynyl)-4-methylbenzene **2b** (Table 1, entry 2)^1^

^1^H NMR (400 MHz, DMSO-*d*_6_) δ 7.64 – 7.56 (m, 1H), 7.30 – 7.19 (m, 1H), 7.22 – 7.15 (m, 1H), 7.11 – 6.98 (m, 1H), 3.57 (s, 1H).

^13^C NMR (101 MHz, DMSO-*d*_6_) δ 136.6, 129.9, 127.6, 117.2, 116.6, 116.2, 82.0, 78.2, 19.3.

FTIR (cm^-^1), 2106 w (C≡C stretch), 1506 m (C=C stretch), 1508 m (C=C stretch), 734 s (C-H bend).

MS (EI^+^) (*m/z*): [M]^+^, 241.9, C_9_H_7_I requires [M]^+^, 241.9.

1-Fluoro-4-(iodoethynyl)benzene **2c** (Table 1, entry 3)^2^

^1^H NMR (400 MHz, DMSO-*d*_6_) δ 7.74 – 7.61 (m, 1H), 7.53 – 7.38 (m, 1H), 7.42 – 7.29 (m, 1H), 7.22 – 7.03 (m, 1H).

^13^C NMR (100 MHz, DMSO-*d*_6_) δ 163.8, 161.3, 134.4, 118.6, 116.3, 82.8, 80.6, 80.6.

FTIR, 2108 w (C≡C stretch), 1599 s (C=C aromatic), 1501 m (C=C aromatic stretch), 1508 m (C=C aromatic stretch), 734 s (C-H bend).

MS (EI^+^) (*m/z*): [M]^+^, 245.9, C_8_H_4_FI requires [M]^+^, 245.9.

4-(Iodoethynyl)benzaldehyde **2d** (Table 1, entry 4)^3^

^1^H NMR (400 MHz, DMSO-*d*_6_) δ 10.05 (s, 1H) 7.89– 7.66 (m, 2H), 7.62 – 7.60 (m, 2H).

^13^C NMR (101 MHz, DMSO-*d*_6_) δ 190.9, 134.2, 130.8, 127.9, 125.9, 116.4, 116.1, 82.2, 81.9.

FTIR, 2100 w (C≡C stretch), 1703 m (C=O), 1681 m (C=O stretch), 1602 w (C=C aromatic), 1473 w (C=C aromatic stretch), 736 m (C-H aromatic bend).

MS (EI^+^) (m/z): [M]^+^, 256.9, C_9_H_5_IO requires [M]^+^, 256.9.

1-(Iodoethynyl)-4-nitrobenzene **2e** (Table 1, entry 5)^1^

^1^H NMR (400 MHz, DMSO-*d*_6_) 8.29 – 8.12 (m, 1H), 7.85 – 7.62 (m, 1H), 7.50 – 7.31 (m, 1H), 7.29 – 7.10 (m, 1H)

^13^C NMR (101 MHz, DMSO-*d*_6_) δ, 137.6, 136.5, 133.7, 131.1, 130.1, 128.9, 124.2, 95.3.

FTIR, 2109 w (C≡C stretch), 1593 w (C=C aromatic), 1508 m (C=C stretch), 734 s (C-H bend).

MS (EI^+^) (*m/z*): [M]^+^, 272.9, C_8_H_4_INO_2_ requires [M]^+^, 272.9.

3-(Iodoethynyl)phenol **2f** (Table 1, entry 6)

^1^H NMR (400 MHz, DMSO-*d*_6_) δ 7.68 (m 1H), 7.43 – 7.26 (m, 2H), 7.22 – 7.02 (m, 1H).

^13^C NMR (101 MHz, DMSO-*d*_6_) δ 156.8, 129.3, 122.1, 122.0, 117.7, 116.0, 83.1, 79.4.

FTIR, 3294 s ( OH stretch), 2102 w (C≡C stretch), 1589 m (C=C aromatic), 1579 m (C=C aromatic stretch), 732 s (C-H bend).

MS (EI^+^) (*m/z*): [M]^+^, 243.9, C_8_H_5_IO requires [M]^+^, 243.9.

NMR Spectra of Compounds

Entry 1 (2a)

Entry 1 (2a)

Entry 2 (2b)

Entry 2 (2b)

Entry 3 (2c)

Entry 3 (2c)

Entry 4 (2d)

Entry 4 (2d)

Entry 5 (2e)

Entry 5 (2e)

Entry 6 (2f)

Entry 6 (2f)

**Metabolic Experimental Procedure**

An aliquot (50 µL) of Gibco pooled human Microsomes (50 donors) (20 mg/mL) was placed into a PTFE tube. 50 µL of 100 mM KH_2_PO_4_ buffer pH 7.4 was added so that concentration was 10 mg/mL. The alkyne (2µM) was dissolved in DMSO (1mL). 20 µL of the alkyne solution was added to 180 µL of 100 mM KH_2_PO_4_ buffer pH 7.4 (6 x of these were made). The 6 solutions were warmed to 37^o^C for 10 mins. 20µL of NADPH 13mM in 100mM KH_2_PO_4_ buffer solution pH 7.4 was added to each of the solutions. Incubations were then terminated at 0, 5, 15, 30, 45, 60 mins by addition of 200 µL ice cold methanol. The solutions were then centrifuged at 1000g for 5 mins at room temperature and 350 µL of supernatant was removed. Each supernatant was diluted with 200 µL methanol. Each solution was then passed through 0.22 µm filter. 50µL of each filtered solution was analysed with the HPLC method described. Controls were as follows;

.

**Control 1:** The alkyne (2µM) was dissolved in DMSO (1mL), alkyne (20µL), buffer 180µL (prepared as above) and ice-cold methanol 600 µL.

**Control 2:** Microsomes 10µL (prepared as above), buffer (prepared as above), 190µL, ice-cold methanol 600 µL.

**Control 3:** NADPH, 20µL (prepared as above), buffer180µL (prepared as above), ice cold methanol 600 µL.

**Control 4:** The alkyne (2µM) was dissolved in DMSO (1mL), alkyne (20µL), microsomes 10µL (prepared as above), buffer 170µL (prepared as above), (incubated at 37^o^C 10 mins), ice cold methanol (600 µL) was then added.

**HPLC Procedure for Metabolism Studies**

A HPLC method was developed using an Agilent Eclipse XDB-C18 5µm, 9.4 x 250mm column. A Phenomenex Security Guard was used, Gemini C18 10 x 10 mm. Mobile phase consisted of A: H_2_O & 1 % Formic Acid, B: MeOH & 1% Formic Acid. Gradient elution (3 mL per min) of A: 40%- 20%, 0-25 mins, A: 20% - 40% 25-30 mins, A: 40% 30-35 mins.

**Synthesis Procedure for Iodoethynyl βAG-TOCA**

# A solution of sodium acetate buffer (pH 5.0, 250 mM) was prepared. To 50 µL of sodium acetate buffer solution was added a solution of copper (II) sulfate pentahydrate (4 eq) BPDS was added (5 eq), in 25 µL of water. Potassium iodide (1 eq) in water was added to this solution followed by βAG TOCA (0.5 µMol, 1eq). The reaction was agitated for 2 hours at ambient temperature. After this time the reaction solution was diluted with water 0.1 % TFA (100 µL) and purified using reverse phase preparative HPLC. HPLC fractions were concentrated by rotary evaporator. Water and formic acid were removed by lyophilization, iodoethynyl βAG-TOCA were then recovered.

**HPLC Procedure for Iodoethynyl βAG-TOCA**

Agilent Eclipse XDB-C18 5µm, 9.4 x 250mm column. A Phenomenex Security Guard was used, Gemini C18 10 x 10 mm. Mobile phase consisted of A: H2O & 1 % Formic Acid, B: MeOH & 1% Formic Acid. Gradient elution (3 mL per min) of A: 40%- 20%, 0-8 mins, A: 20% - 40% 8-15 mins, A: 40% 15-20 mins.


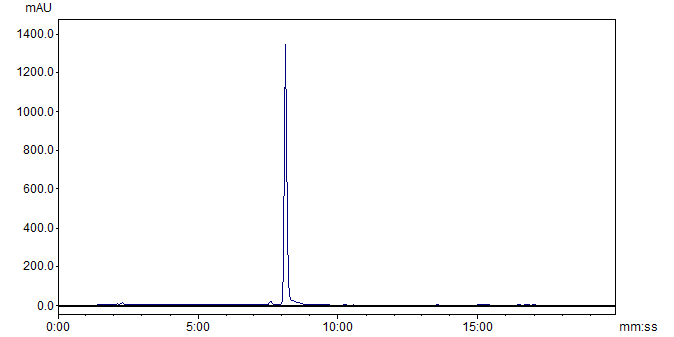
HPLC Chromatogram of Iodoethynyl βAG-TOCA

**NMR Spectra of Iodoethynyl** **βAG-TOCA**

**Mass Spectrometry Data for Iodoethynyl ΒAG-TOCA Derivative**


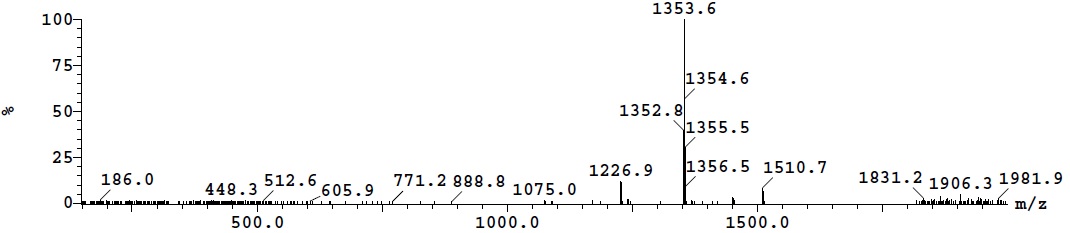


MALDI TOF spectra of Iodoethynyl βAG-TOCA

**Radiolabelling Procedure for 2a**

# To acetonitrile (100 μL) was added the alkyne **1a** (3 mg). Bathophenanthrolinedisulfonic acid disodium salt hydrate (BPDS) (6.6 mg) was dissolved in purified water (100 µL) and added to the alkyne solution. Radioactive potassium iodide-125 (KI), (50-100 μCi per attempt) was added to the solution and subsequently, a solution of sodium acetate buffer (pH 5.0, 250 mM, 100 μl) was added, containing copper (II) sulfate pentahydrate (3 mg) was then added to the alkyne solution. The reaction was left at room temperature for 30 minutes, before a sample was taken (5-10 μl) and diluted in water (2 ml). RadioHPLC was carried out (using a water:MeCN gradient of 95-5 over 15 minutes), giving a R_t_ = 6.0 minutes (which was collected from the HPLC to give an accurate determination of the yield), whilst iTLC was performed on silica TLC plates run in 95:5 water:MeCN, giving a radiochemical yield of 16 % (n = 3).

**References**

[1] O. Dumele, D. Wu, N. Trapp, N. Goroff and F. Diederich, *Org. Lett.*, 2014, **16**, 4722–4725.

[2] J. García-Álvarez, J. Diez and J. Gimeno, *ChemInform Abstract: A Highly Efficient Copper(I) Catalyst for the 1,3-Dipolar Cycloaddition of Azides with Terminal and 1-Iodoalkynes in Water: Regioselective Synthesis of 1,4-Disubstituted and 1,4,5-Trisubstituted 1,2,3-Triazoles.*, 2011, vol. **42**.

[3] W. H. Ojala, T. M. Arola, A. M. Brigino, J. D. Leavell and C. R. Ojala, *Acta Crystallogr. Sect. C*, 2012, **68**, o270–o278.
